# The Effect of the Body Mass Indexes of Young Healthy Individuals on the Glyacemic Indexes of Traditional and Modified Vegetarian Meals

**DOI:** 10.3390/nu11102546

**Published:** 2019-10-22

**Authors:** Ewa Raczkowska, Monika Bronkowska

**Affiliations:** Department of Human Nutrition, Faculty of Biotechnology and Food Sciences, Wrocław University of Environmental and Life Sciences, 51-630 Wrocław, Poland; monika.bronkowska@upwr.edu.pl

**Keywords:** glycaemic index, vegetarian meals, BMI

## Abstract

Blood glucose concentration increases after the consumption of any carbohydrate-containing meal. Several factors affect the course of glucose metabolism, including nutritional status. This study evaluated the effect of the nutritional statuses of adults on their glycaemic responses after the consumption of some vegetarian meals (dumplings with potato and curd cheese stuffing; curd cheese dumplings; pancakes with curd cheese), prepared according to the traditional recipe and a partly modified recipe. The 105 participants, aged 20–27 years, with different body mass indexes (BMI), took an oral glucose tolerance test after the intake of a standard glucose solution, and also after each meal (previously analysed for energy value and approximate composition). The consumption of each meal by participants with different nutritional statuses elicited different glycaemic responses, which were reflected in the diverse glycaemic indexes (GIs). The partial modification of the meal recipes contributed to lowering their GIs. Vast differences were observed in the glycaemic responses among the surveyed participants after the consumption of the same meals. The GIs of meals should be determined in different groups of people.

## 1. Introduction

The glycaemic index (GI) is applied worldwide as a physiological factor, which differentiates food products that contain carbohydrates by how they affect the postprandial blood glucose concentration [1]. It has been popularised as a fundamental tool in the choice of food products aimed at reducing the risk of development of obesity, diabetes, and cardiovascular diseases. The knowledge of the GIs of food products allows choosing foods that stimulate insulin secretion and avoiding food products that enhance insulin resistance [2,3,4].

Many factors, including the body mass index (BMI), influence glucose’s metabolism in humans. An increased content of adipose tissue contributes to the loss of tissue sensitivity to insulin. The fasting glucose concentration and postprandial glycaemia are higher in individuals with overweight or obesity than those with normal BMIs [5]. Agrawal et al. [6] evaluated the effect of BMI on fasting glucose levels in a group of 150 people aged 20–70 years and observed a significant, positive correlation between BMI and fasting glucose concentration (*r* = 0.751, *p* < 0.0001). Ritchie and Connell [7] concluded that obesity and the adiposity of internal organs were key factors in the development of tissue resistance to insulin because an increase in the content of visceral fatty tissue contributed to dyslipidaemia, the enhancement of gluconeogenesis, and insulin resistance. Snijder et al. [8,9] proved that an increased postprandial insulin concentration in men was due to excessive visceral obesity, while in women, the peripheral distribution of fatty tissue was linked to increased insulin sensitivity.

The phenotype of obesity is also of great significance. In a study of 43 women aged 52–64 years and affected with obesity, Brochu et al. [10] found that 17 women had a normal metabolic profile but higher insulin sensitivity. The increased insulin sensitivity was associated partly with having lower visceral fat (49% less) and an earlier age-related onset of obesity compared with those with metabolically normal obesity. The studies mentioned above indicate that people with relatively higher BMIs are characterised by corresponding increases in insulinaemia values. Some of the above-cited investigations suggest that the successive increase in visceral fatty tissue content, which induces insulin resistance, is the key pathogenetic factor responsible for the development of carbohydrate metabolism disorders.

Several factors affect the GI value of food. Some of these factors are the degree of product processing, the type of heat treatment, the carbohydrate profile of the food, the structure of starch, and the presence of other nutrients, such as proteins, fats, dietary fibre, and organic acids [11,12,13,14]. In the face of rising concerns in obesity and obesity-related chronic diseases, the challenge for the food industry is to modify the composition of high GI foods, while maintaining high nutritional value. A balanced diet is an important factor in maintaining good health and potentially reduces the risk of disease [15,16,17]. Vegetarian dishes are often modified to confer them with additional health benefits. The most common modification is to replace wheat flour with whole-grain flour or add bran from different cereals, which increases the contents of dietary fibre and minerals [18].

The main objective of this study was to evaluate the effects of the nutritional statuses of young, healthy individuals on their glycaemic responses after the consumption of some vegetarian meals typical of Polish cuisine, prepared according to traditional recipes and partly modified recipes.

## 2. Materials and Methods

### 2.1. Determination of the Nutritional Value of the Analysed Meals

#### 2.1.1. Recipes and Preparing the Meals

The GIs were determined for three typical vegetarian meals: dumplings with potato and curd cheese stuffing; curd cheese dumplings; and pancakes with curd cheese. Both dumpling dishes are frequently eaten as dinners, whereas the pancake with cheese dish is popular as a breakfast and dessert. Two versions of each dish were prepared: one traditional recipe and one partly modified recipe.

Dumplings with potato and curd cheese stuffing—traditional version.

Raw material composition:

- Curd cheese, fatty—34 g;
- Chicken eggs, whole—4 g;
- Butter—10 g (5 g for stuffing, 5 g for direct consumption);
- Wheat flour type 471—54 g (18 g for brewing);
- Onion—30 g (15 g for stuffing, 15 g for direct consumption);
- Potatoes—110 g;
- Water—30 g;
- Cake salt—0.07 g;
- Salt for stuffing—0.7 g;
- Pepper for stuffing—0.12 g.

Method of implementation:

Potatoes were cooked in unison, cooled, peeled, and blended with the cottage cheese in a KitchenAid (USA) multi-function electric machine. Onions were diced, and 15 g was fried for 3 min with 5 g of butter and added to the potatoes and cheese. The stuffing was mixed thoroughly and seasoned with salt and pepper. The first stage of dough preparation consisted of brewing 1/3 of the quantity of wheat with hot water. The remainder of the flour, eggs, and salt were then added, and a homogenous dough was formed. After the dough was left covered for 30 min, it was rolled out. Rings were cut out of the dough, which was stuffed and formed into dumplings by gluing the edges of the dough. The dumplings were cooked in boiling water for 4 min, including 2 min after the dumplings came to the surface. Dumplings were served with onion fried in butter for 3 min (15 g of onion and 5 g of butter).

Curd cheese dumplings—traditional version

Raw material composition:

- Curd cheese, lean cheese—100 g;
- Chicken egg, yolk—10 g;
- Butter—5 g;
- Wheat flour type 471—50 g;
- Bread crumbs—6 g;
- Icing sugar—12 g (7 g for the dough, 5 g for direct consumption).

Method of implementation:

Cottage cheese was ground in a KitchenAid (USA) multi-function electric machine, and then yolks, sugar, and wheat flour were added, and the dough was formed. A roll was formed from the dough, slightly flattened and cut into diagonal pieces. Curd cheese dumplings were boiled for 4 min in boiling water (until they emerged on the surface). The dumplings were served with breadcrumbs, butter and icing sugar.

Pancakes with curd cheese—traditional version:

Raw material composition:

- Milk, 2% fat—55 g;
- Curd cheese, fatty—90 g;
- Chicken eggs, whole—15 g (added to the dough);
- Chicken egg, yolk—10 g (added to the stuffing);
- Oil—3 g;
- Butter—4 g;
- Wheat flour type 471—50 g;
- Icing sugar—14 g (10 g for stuffing, 4 g for direct consumption);
- Salt—0.05 g;
- Water—45 g.

Method of implementation:

The milk, water, eggs, oil, wheat flour, and salt were mixed with a KitchenAid (USA) multi-function mixer to a homogenous mass. A small amount of dough was poured into a heated frying pan, and the pancakes were fried after 1 min on either side. The curd cheese was ground in a KitchenAid (USA) multi-functional electrical machine; egg yolks and icing sugar were added and mixed thoroughly. Pancakes were stuffed and folded into triangles. The pancakes were fried in butter for 50 s on each side and served sprinkled with the remaining icing sugar.

The modification of meals included substituting type 471 wheat flour with type 1630 wholegrain wheat flour (as the type of flour was not declared by the producer, it was determined analytically by assaying the ash content).

#### 2.1.2. Nutritional Value Analysis

All meals were hand-made in a gastronomic unit of the Department of Human Nutrition at the Wrocław University of Environmental and Life Sciences (Wrocław, Poland). To determine nutritional value, each meal was prepared in two portions, each containing 50 g of available carbohydrates. The meals were homogenised and analysed for energy (Rozental’s method) [19]. The Rozental method is based on the oxidation of the sample with potassium dichromate in the medium of sulphuric acid. The energy value of the product was then calculated using the experimentally determined coefficient. Standard AOAC methods [20] were used to measure:

- The contents of moisture and dry matter (gravimetric-drying method). The method consisted of drying the specimens, first by pre-drying at 60 °C. The food samples were mixed with roasted sand. This increases the evaporation surface. The samples were dried at 105 °C to constant weight.
- Total ash: The method consisted of determining the mass of the residue after ashing the samples. The combustion of the sample was carried out in two-step process: carbonization of the sample and roasting in a muffle furnace at 600 °C.
- Protein (Kjeldahl method): The method consisted of the mineralization of the tested substance in boiling sulphuric acid which resulted in proteins being oxidized to CO_2_ and H_2_O. Nitrogen from amino protein groups was released in the form of ammonia and it bound to sulfuric acid. After alkalising the sample, the ammonia was distilled to boric acid and then titrated with a standard solution of hydrochloric acid.
- Fat (Soxhlet method): The method consisted of an extraction with ethyl ether. The solvent was then evaporated, and the residue weighed.
- Dietary fibre (enzymatic-gravimetric method): The method consisted of etching the degreased sample successively with thermo-resistant α-amylase, protease, and amyloglucosidase. After precipitation of the soluble fibre, the undigested parts were determined by weight.

For the content of available carbohydrates per serving of each dish, the difference between the dry matter content and the sum of the contents of ash, fat, protein, and dietary fibre was calculated.

The energy value and approximate composition of the analysed meals provided in a portion that provides 50 g of available carbohydrates, and those based on the actual serving size, are given in Supplementary Table S1.

### 2.2. Study Design and Participant Characteristics

This study was conducted at the Department of Human Nutrition of the Wrocław University of Environmental and Life Sciences and approved by the Bioethical Commission of the Medical University in Wrocław (approval number KB–831/2012).

#### 2.2.1. Stage I: Recruitment

**Figure 1:**
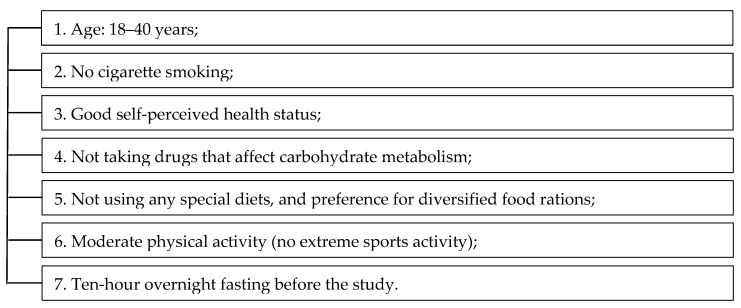
Inclusion criteria for the participation in the study.

The participants were recruited from the Wrocław University of Environmental and Life Sciences, following an oral presentation of the study’s purpose and conditions, and the course of the research. Each individual gave written consent to participate in the study (165 people registered for the study). About 30 people meeting all the criteria were recruited for each BMI group (Figure 1).

#### 2.2.2. Stage II: Anthropometric Measurements and Filling in the Questionnaire

The nutritional status of the surveyed participants was evaluated based on anthropometric measurements of body mass (±0.01 kg) in fasted participants, and body height (±0.1 cm) determined using an electronic medical scale with a stadiometer (Radwag, Radom, Poland). During the measurements, each person was shoeless, without outer clothing and was asked to stand up straight. BMI was calculated as body mass (kg) divided by the square of body height (m^2^).

Each participant completed a short questionnaire to obtain information about health status, eating habits, smoking, medications/hormone intake, and physical activity.

Both sets of data were collected in 1 week, and 165 participants took part. After receiving the participants’ responses to the questionnaire, 20 individuals were excluded due to diagnosed metabolic diseases and/or cigarette smoking and/or competitive sports and/or drugs/hormones that may have affected carbohydrate metabolism. A total of 145 participants qualified for stage III of the screening process.

#### 2.2.3. Stage III: Completion of 24-h Dietary Diaries

Participants completed a 3-day food diary, which was analysed for dietary habits, special diets, and preferences concerning different dietary rations. Meetings to discuss dietary diaries in detail were held within 1 week. Based on the data, 18 patients were excluded due to special diets (e.g., mono-diets). A total of 127 participants qualified for Stage IV of the screening process.

#### 2.2.4. Stage IV: Fasting Glucose Measurement

Fasting glucose measurements took place over 3 days (Monday, Wednesday, and Friday). A further 22 participants were excluded due to fasting glucose concentration >99.0 mg/dL (*n* = 11) and unavailability (*n* = 11). Finally, 105 participants qualified for the study and were divided into three BMI (kg/m^2^) groups: <18.5 (*n* = 32), 18.5–24.9 (*n* = 34), and ≥25.0 (*n* = 39).

#### 2.2.5. Stage V: Oral Glucose Tolerance Test (OGTT)

At baseline and within a week after administration of all test meals, a standard OGTT was performed by dissolving 50 g of crystalline glucose in 250 cm^3^ of warm boiled water, just before it was served to the participants. The participants had 5–10 min to drink the glucose solution. Capillary blood samplings were taken from a fingertip using an automatic Accu-Chek Softclix lancing device (Roche Diagnostics, Rotkreuz, Switzerland) at 15, 30, 45, 60, 90, and 120 min for the measurement of blood glucose concentration (Accu-Chek Active glucometer, Roche).

Participants’ baseline data and characteristics are summarised in Table 1. All participants had secondary education as the highest qualification. All measurements were made at the Wrocław University of Environmental and Life Sciences. Each participant was met eight times at 7-day intervals. All participants were instructed to continue their eating habits and their normal physical activity throughout the study, but to minimise the intake of alcohol, caffeine-containing beverages, and intensive physical activity a day before the OGTT and the administration of test meals, which were scheduled as shown in Table 2. All data were recorded in Microsoft Excel 10 datasheets prepared for each participant.

### 2.3. Administration of Test Meals and GI Determination of Meals

Twice during the two weeks following the baseline OGTT, participants returned after an overnight fast of at least 10-h, for measurement of their blood glucose concentrations (see Section 2.2.5) before and at 15, 30, 45, 60, 90, and 120 min after the beginning of the consumption of the test meals, which occurred during the morning hours of working days (Table 2). The meals were prepared at the study site and served to the participants on the same day, on uniform, white, disposable plates. The participants had 10–15 min to eat the meals.

The GI of each meal was determined by dividing the area under the curve plotted for each analysed meal by the area obtained for the glucose solution, and multiplying the result by 100, in compliance with the ISO 26642:2010 standard [21] and FAO/WHO recommended procedures [22]. The GI of each meal is an arithmetic mean of GI values computed individually for each meal for each participant. The size of the area under the glycaemic curves was computed for each person after consumption of each meal and glucose solution, by dividing the area into triangles and trapezoids. Negative values of area size were not taken into account during calculations.

### 2.4. Statistical Analysis

Data were statistically analysed using Statistica version 12.5 PL (StatSoft, Tulsa, OK, USA). The Shapiro–Wilk test was used to determine the normal distribution of the variables. In most cases, the data were not normally distributed, so the results were expressed as the median and lower and upper quartile values. For non-normally distributed data, the Box–Cox transformation was performed to normalise the data. The Student’s *t*-test, for independent variables, was applied to demonstrate significant differences between the GI values determined for the meals made according to the traditional and partly modified recipes, respectively. Differences in GIs, areas under glycaemic curves, and glycaemic responses after the consumption of traditional and modified vegetarian meals, as affected by the BMI of the study participants, were determined by one-way analysis of variance (ANOVA), followed by Tukey’s test, for multiple comparisons between groups. Values of the coefficient of variability (CV) of glycemia after 2-fold intake of the standard glucose solution did not exceed 15% (the required CV is <30%). The GI values of a given meal calculated for individuals that exceeded twice or more the standard deviation in a given group were eliminated from further analyses.

## 3. Results

### 3.1. Glycaemic Responses to OGTT and the Tested Meals 

**Figure 2:**
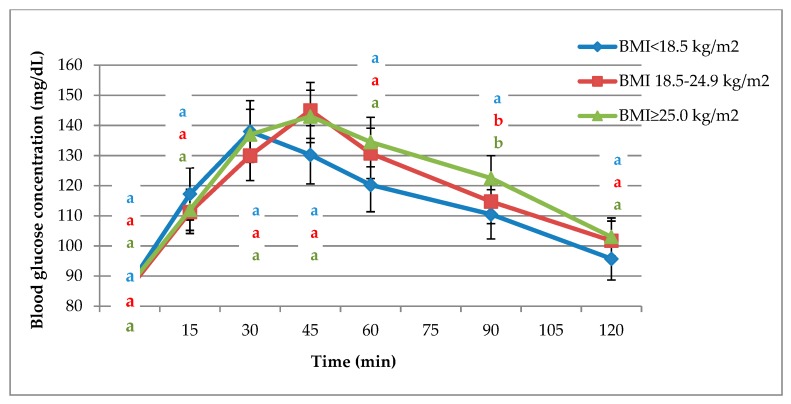
Curves of glycaemic response after consumption of a standard glucose solution. (**a**,**b**): significantly different at *p* < 0.05.

The glycaemic response curves elicited after consumption of the standard glucose solution are presented in Figure 2 for all participants. The highest glycaemic response was observed in the group with BMI ≥ 25.0 kg/m^2^. The maximum glucose concentration in participants with BMI ≥ 18.5 kg/m^2^ was obtained in the 45th min of the test compared with the 30th min for the group with BMI < 18.5 kg/m^2^. BMI had no significant influence on fasting glucose concentration. Regardless of the BMI, the glucose concentration in each measurement during 2 h did not differ significantly, except at 90 min. The blood glucose concentration was significantly lower in participants with a BMI < 18.5 kg/m^2^ than those with normal BMIs and BMIs ≥ 25.0 kg/m^2^.

The median blood glucose concentrations within 2 h since the consumption of traditional and modified meals and the areas under the glycaemic curves are presented in Supplementary Tables S2–S4. After consuming dumplings with potato and curd cheese prepared by the traditional recipe, participants with BMIs < 18.5 kg/m^2^ and BMIs ≥ 25.0 kg/m^2^ showed the highest blood glucose concentrations at 30 min of the test, with values of 142.0 and 111.0 mg/dL, respectively. In participants with normal BMIs, the highest blood glucose concentration occurred at 45 min and reached 122.0 mg/dL. After the consumption of the modified version of this meal, regardless of the BMI, the highest blood glucose concentration was elicited at 45 min. Modification of this meal recipe contributed to a significantly lower glycaemic response after its consumption by those participants with BMIs < 18.5 kg/m^2^, throughout the 2 h, and a significant reduction in the area under the glycaemic curves (Supplementary Table S2).

Following the consumption of both versions of curd cheese dumplings, the highest glucose concentration was elicited at 30 min. An exception was the glucose concentration noted after consumption of the modified dumplings by the participants with normal BMIs, whose highest value occurred at 45 min and reached 110.0 mg/dL. Modification of this recipe contributed to a significant reduction in the areas under the glycaemic curves (Supplementary Table S3).

After the consumption of traditional and modified pancakes with curd cheese, the highest blood glucose concentrations appeared at 45 min, among all three groups of participants. There were no significant differences in the glycaemic responses within 2 h after the consumption of traditional and modified pancakes with curd cheese, due to the BMIs of the participants. In addition, the modification of the recipe for pancakes with curd cheese caused a significant reduction in the areas under the glycaemic curves. After consumption of the traditional version of the meal, the participants with BMIs 18.5–24.9 kg/m^2^ displayed a significantly higher area under the glycaemic curve in comparison to the other groups (Supplementary Table S4).

### 3.2. GI of the Tested Meals

The GI values of the analysed meals are presented in Supplementary Table S5. The partial modification of the recipes of all meals contributed to a decrease in their GIs. A comparison of the GIs of meals made according to the traditional and modified recipes among participants with BMIs < 18.5 kg/m^2^ demonstrated that for dumplings with potato and curd cheese stuffing, the modification of the recipe caused a significant decrease in the GI. The GI values of all traditional meals consumed by participants with normal BMIs were significantly higher than the GI values of the partly modified meals. In turn, a comparison of the GI values of meals consumed by participants with BMIs ≥ 25.0 kg/m^2^ revealed a significant decrease in the GI of pancakes with curd cheese prepared by the partly modified recipe.

## 4. Discussion

Modification of the recipes for the examined dishes caused changes in nutritional value and differences in glycaemic responses after their consumption. Modified dishes were characterised by lower GIs compared to traditional dishes. The effect of BMI on blood glucose levels was also observed after the consumption of vegetarian dishes. It was observed that portions of modified dishes contained significantly more dietary fibre, fat, protein, and total carbohydrates in comparison to the traditional versions (Supplementary Table S1). Dietary fibre is an essential component of the human diet due to its health benefits [23,24,25]. Călinoiu and Vodnar [26] showed that wholegrain flour products have excellent nutritional properties due to their pro-health nutrients. An inverse correlation was found between the consumption of wholegrain products and the risk of chronic diseases and metabolic syndromes [26]. In addition, dietary fibre, fat, and protein markedly lower the GIs of food products, as confirmed, amongst other studies, by the research of Kurek et al. [27]. The present investigation led to a similar conclusion. Modified foods with higher fibre, fat, and protein contents than their traditional counterparts, also had lower GIs (Supplementary Table S5).

The course of the glycaemic curves after consumption of the reference glucose solution is shown in Figure 2. In this study, as well as that of Matthan et al. [28], BMI did not significantly affect glucose concentration, except at 90 min. Blood glucose concentrations in participants with BMIs < 18.5 kg/m^2^ were significantly lower than in the other two groups (Figure 2).

Dolna et al. [29] recruited 14 young, healthy individuals with an average age of 26.5 years to determine the GI of selected vegetarian meals typical of Polish cuisine: pancakes with curd cheese, beans stewed with meat in tomato sauce, dumplings with curd cheese, vegetable salad, vegetable soup, and carrots with peas. In that research, the maximal blood glucose concentration was observed at 30 min after consumption of curd cheese dumplings and the pancakes with curd cheese. In our own study, however, it was after 30 and 45 min, respectively (Supplementary Tables S3 and S4). 

According to the international standard for determining GI (ISO 26642:2010) [21], most of the traditional meals and all analysed meals prepared by the partly modified recipe could be classified as low-GI products (GI ≤ 55.0%) (Supplementary Table S5). In our participants, the GI of pancakes with curd cheese, determined in the group with a normal BMI was 53.4%, and was 61.0% for curd cheese dumplings (Supplementary Table S5), compared to 35.0% and 12.6%, respectively, reported by Dolna et al. [29]. Differences in GI values may result from the different proportions of flour, sugar, and butter in the recipes. In addition, their portion of dumplings was prepared from 240 g of full-fat cheese [29], whereas, in our study, it was prepared from 100 g of skimmed-milk cheese. The content of full-fat cheese in these meals, as well as the amount of fat used for frying the pancakes and glazing the curd cheese dumplings, significantly lowered the GI, despite the presence of wheat flour and sugar in the recipe [11,30].

Variations in GIs were also significantly influenced by differences in the metabolism among the participants. Comparing the BMIs of individuals in this study, significant differences in GIs were observed only after consumption of the modified version of dumplings with potatoes and curd cheese stuffing (*p* = 0.014) (Supplementary Table S5). Wolever et al. [31] demonstrated large differences in the GIs of five food products determined by various research centres. All of the seven laboratories from Canada, Sweden, Australia, Italy, New Zealand, West Indies, and South Africa determined the GI of each food product (instant potato, rice, spaghetti, white bread, and barley) by following the WHO/FAO recommended procedure. Notably, the authors stated that when GI values differ by more than 18 for the same food, as analysed by reputable research centres, the findings represent genuine differences in GI rather than serendipitous results. It was further mentioned that the exact reasons for the discrepancies in the GI were unclear because differences in the metabolic processes of the surveyed participants caused by their various origins, the type of blood sampled (capillary or vascular), or the measurement methods, for instance, could have been contributing factors [31]. Venous blood has a lower glucose concentration relative to capillary blood, because as blood flows from the arterial to the venous circulation via the capillaries, peripheral tissues remove some of the glucose. The concentrations of glucose within blood varies too. Whole blood glucose levels are lower than plasma levels and this is due to differences in water content between whole blood and plasma. The rise in blood insulin and glucose after eating stimulates glucose removal by tissues. Blood glucose levels are conditioned by pulsed insulin secretion. The oscillations of plasma glucose in different tissues in the body are not in phase with each other, because it takes different lengths of time for the pulses of insulin from the pancreas to reach them. The magnitude of glucose oscillations in forearm venous blood may be greater than those in capillary blood because the vein drains a small volume of tissue with insulin oscillations in phase with each other [31].

Basu et al. [32] applied a multi-way analysis to examine the effect of, among other things, the maximum rate of oxygen consumption and fatness degree on the reduction of glucose tolerance with age. Peak oxygen uptake (an index of aerobic fitness) was measured using a standard treadmill stress test. Peak oxygen uptake was lower (*p* < 0.001) in the elderly than in the young participants. Univariate analyses indicated that insulin action was significantly correlated with percentage of body fat (*r* = −0.60; *p* < 0.001), visceral fat (*r* = −0.35; *p* < 0.001), peak oxygen uptake (*r* = 0.36; *p* < 0.001), and fasting glucose (*r* = −0.31; *p* < 0.01) It was observed that insulin concentration in the fasted state and after the OGTT in individuals characterised by both a high content and unfavourable distribution of adipose tissue in the body was closely correlated with the impairment of insulin action and secretion [32].

Elastase-1 is a glycoprotein with enzymatic properties and is resistant to degradation by bacterial flora. Its concentration in the faeces typically reaches >200 μg/g. A concentration in the range 100–200 μg/g is indicative of mild and moderately acute pancreatic failure, while concentrations < 100 μg/g are indicative of acute exocrine pancreatic insufficiency [33]. Teichmann et al. [34] showed that the concentration of elastase-1 in faeces of women with BMIs > 30 kg/m^2^ was lower compared with women with normal BMIs. Its low concentration was not affected by disorders in carbohydrate metabolism, and disorders in the functions of the pancreas and gallbladder.

Obesity is most frequently accompanied by an inflammatory state, which entails enhanced release of pro-inflammatory molecules, like monocyte chemoattractant protein-1, tumour necrosis factor-α, hepatocyte growth factor, plasminogen activator inhibitor-1, interleukin-6, and interleukin-8, and the suppressed synthesis of adiponectins. A high concentration of triglycerides in the blood of obese rats contributes to greater impairment of pancreas function in the course of the inflammatory process. High body weight is additionally linked with excess production of reactive oxygen species, which cause damage to cells and tissues, and are involved in various pathologies [35]. Excessive body mass is a prognostic factor for determining the extent of acute pancreatitis in rats. Pancreatitis is facilitated by a high concentration of isoprostanoids (compounds formed by a non-enzymatic oxidation of polyunsaturated fatty acids) and is accompanied by the formation of necrotic foci. Pancreatic lipase has a key role in necrosis development. This enzyme shows high affinity for the white adipose tissue at the simultaneous intensification of its necrosis [35].

Results of investigations conducted so far confirm that obese individuals tend to accumulate fatty tissue in the liver and muscles [36,37]. An investigation conducted by Lê et al. [38] also confirmed a positive, linear correlation of pancreatic fat deposition with BMI and age. Pancreatic fat deposition was closely linked to additional deleterious fat depots, including visceral adipose tissue and liver fat, and was positively associated with circulating free fatty acids. It was further noted that ethnic differences in pancreatic fat deposition are exacerbated by age, even in young populations. Hispanics accumulate more pancreatic fat than African Americans and are at high risk for metabolic disease. Fatty tissue content in the pancreas is linked to reduced insulin resistance and suppressed insulin secretion. Glucose tolerance decreases with age because of a decline in both insulin secretion and action. As explained by Basu et al. [32], the extent of this insulin defect is linked to the physical aspects of ageing (increases in the percentages of body fat and visceral fat) rather than age per se.

Few works to date have modified the recipes of meals, including the replacement of wheat flour with whole-grain wheat flour, and investigated the effect of these modified products on postprandial glycaemia [39,40,41]. A related investigation was conducted by Bae et al. [39], but it aimed at determining the effect of whole-grain flour on dough’s quality and GI, estimated based on the rate of starch digestion by an in vitro method. In that study, the dough samples prepared from whole-grain flours (wheat and buckwheat) had significantly lower GIs, and dough samples with 50% addition of whole-grain flours maintained desirable volumes and structures. Ferrer-Mairal et al. [40] evaluated the GIs of bread and muffins using two different in vivo methods and an in vitro assay of the rate of starch digestion. As a result, the partial replacement of wheat flour with a mixture of resistant starch, non-digestible dextrin, and lentil flour significantly decreased the GIs of the studied bakery products. Raczkowska et al. [41] examined the GIs of selected dishes prepared from yeast dough based on their traditional and modified recipes, in which wheat flour (type 500) was replaced with whole-wheat flour (type 2000). Similar to the present study (Supplementary Table S5), the modification of the formulas increased the protein and fibre contents (Supplementary Table S1) and decreased the GI [41], which positively affected postprandial glycaemia.

So far, the GI values have been designated only with the participation of young, healthy people with normal BMIs. It is necessary to extend the work to ascertain the GIs for people of different ages and nutritional statuses, due to large differences in glycaemic responses after eating the same food. GI values designated only with the participation of young, healthy people with normal BMI are not equivalent and should not be the basis for the planning of nutrition for all groups of people.

## 5. Conclusions

The modification of three vegetarian recipes by replacing wheat flour (type 471) with whole-grain wheat flour (type 1630) significantly increased the fibre, fat, protein, and total carbohydrate contents. The BMI of people aged 20–27 is a significant factor influencing the course of glycaemic curves. BMI significantly affected the GI only for the modified version of dumplings with potato and curd cheese stuffing. For participants with normal BMI, all traditional meals had significantly higher GIs than those of the partly modified meals. It is necessary to determine the GIs of meals in individuals with different nutritional status because of the large differences in glycaemic responses after the consumption of the same meals.

## Figures and Tables

**Table 1:** Baseline clinical and anthropometric characteristics (mean ± standard deviations (SD) of subjects (*n* = 105) involved in the study.

Parameter	BMI < 18.5 kg/m^2^ (*n* = 32)		BMI 18.5–24.9 kg/m^2^ (*n* = 34)		BMI ≥ 25.0 kg/m^2^ (*n* = 39)	
	Mean ± SD	Range	Mean ± SD	Range	Mean ± SD	Range
age (years)	23.70 ± 1.76	20.00–27.00	24.20 ± 0.55	23.00–26.00	23.30 ± 1.12	21.00–26.00
body weight (kg)	48.54 ± 3.07	41.73–54.64	63.54 ± 9.28	49.64–93.92	76.82 ± 10.36	64.05–99.02
height (m)	1.65 ± 0.04	1.55–1.73	1.68 ± 0.09	1.53–1.95	1.66 ± 0.07	1.53–1.84
BMI (kg/m^2^)	17.82 ± 0.59	15.72–18.41	22.3 ± 1.53	20.02–24.83	28.01 ± 3.10	25.04–36.52
fasting glucose (mg/dL)	83.00 ± 0.10	78.00–94.00	81.00 ± 0.20	73.00–88.00	81.00 ± 0.10	78.00–94.00
gender (female/male)	28/4	-	29/5	-	30/9	-
*Place of residence (persons):*						
city ≥ 300,000 inhabitants	25	-	22	-	27	-
city 100,000–300,000 inhabitants	4	-	3	-	2	-
city 10,000–100,000 inhabitants	2	-	5	-	1	-
city ≤ 10,000 inhabitants	0	-	1	-	5	-
village	1	-	3	-	4	-

BMI: body mass index.

**Table 2:** Schedules of the oral glucose tolerance test and the administration of test meals.

Week	Monday	Tuesday	Wednesday
	BMI Group 1 (*n* =32)	BMI Group 2 (*n* = 34)	BMI Group 3 (*n* = 39)
1	OGTT	OGTT	OGTT
2	T1 (*n* = 10), T2 (*n* = 12), T3 (*n* = 10)	T1 (*n* = 10), T2 (*n* = 12), T3 (*n* = 12)	T1 (*n* = 13), T2 (*n* = 11), T3 (*n* = 15)
3	M1 (*n* = 10), M2 (*n* = 12), M3 (*n* = 10)	M1 (*n* = 10), M2 (*n* = 12), M3 (*n* = 12)	M1 (*n* = 13), M2 (*n* = 11), M3 (*n* = 15)
4	OGTT	OGTT	OGTT

BMI (kg/m^2^) was divided into three groups: <18.5 (Group 1), 18.5–24.9 (Group 2), and ≥25.0 (Group 3). OGTT: oral glucose tolerance test. T1, T2, T3, M1, M2, and M3 refer to the traditional (T) and modified (M) versions of dish 1 (dumplings with potatoes and curd cheese), dish 2 (curd cheese dumplings), and dish 3 (pancakes and curd cheese), respectively.

## Supplementary Materials

The following are available online at https://www.mdpi.com/2072-6643/11/10/2546/s1: Table S1: Contents of nutrients and portion size of the analysed vegetarian meals, Table S2: Blood glucose concentration (mg/dL) of the study participants within 2 h after the consumption of dumplings with potatoes and curd cheese stuffing prepared according to the traditional and partly modified recipes, Table S3: Blood glucose concentration (mg/dL) of the study participants within 2 h after the consumption of curd cheese dumplings prepared according to the traditional and partly modified recipes, Table S4: Blood glucose concentration (mg/dL) of the study participants within 2 h after the consumption of pancakes with curd cheese prepared according to the traditional and partly modified recipes, Table S5: Glycaemic indices of meals prepared according to the traditional and partly modified recipes.
