# Supplementary material for: The Effect of the Body Mass Indexes of Young Healthy Individuals on the Glyacemic Indexes of Traditional and Modified Vegetarian Meals
Source: Nutrients. 2019 Oct 22;11(10):2546. doi: 10.3390/nu11102546 (PMC6835997; doi:10.3390/nu11102546)
Supplement: Supplementary file 1 [file nutrients-11-02546-s001.pdf]

nutrients MDPI

Table S1. Contents of nutrients and portion size of the analysed vegetarian meals

Meal Type	Served Portion		Fat		Protein		Total Carbohydrates		Dietary Fibre		Energy Value	
	(g)	*p*	(served portion, g)	*p*	(served portion, g)	*p*	(served portion, g)	*p*	(served portion, g)	*p*	(served portion, g)	*p*
dumplings with potato and curd cheese stuffing – T	264.0	0.068	10.0	0.005	13.3	0.002	52.5	0.001	2.7	<0.001	326.9	0.018
dumplings with potato and curd cheese stuffing – M	322.1		11.9		17.4		58.3		7.7		380.2	
curd cheese dumplings – T	215.0	0.084	7.0	0.002	22.6	<0.001	52.3	0.008	1.7	<0.001	346.7	0.020
curd cheese dumplings – M	217.0		8.9		30.6		55.9		6.3		395.6	
pancakes with curd cheese – T	215.0	0.072	18.3	0.006	22.6	0.006	52.3	0.001	1.7	0.002	412.9	0.017
pancakes with curd cheese – M	246.0		21.1		26.5		55.7		6.1		477.5	

T – traditional version meals, M – modified version meals

Table S2. Blood glucose concentration (mg/dL) of the study participants within 2 h after the consumption of dumplings with potatoes and curd cheese stuffing prepared according to the traditional and partly modified recipes.

**Time (min)**	**BMI < 18.5 kg/m² (*n*=10)**			**BMI 18.5–24.9 kg/m² (*n*=10)**			**BMI ≥ 25.0 kg/m² (*n*=13)**			***p*-BMI T**	***p*-BMI M**
	**T**	**M**	***p***	**T**	**M**	***p***	**T**	**M**	***p***		
0	85.0 (82.0;89.0) **a, A**	89,0 (88.0;94.0) **b, C**	0.008	84.5 (72.0;88.0) **c, A**	87.0 (81.5;90.0) **d, C**	0.023	86.0 (82.0;90.0) **e, A**	85.0 (80.0;89.0) **f, C**	<0.001	0.998	0.955
15	114.0 (99.0;120.0) **a, A**	99.0 (96.0;102.0) **b, C**	0.002	92.0 (89.0;100.0) **c, B**	95.0 (84.0;98.0) **c, C**	0.859	93,0 (86.0;103.0) **e, B**	90.0 (85.0;92.0) **e, C**	0.308	0.030	0.580
30	142.0 (128.0;145.0) **a, A**	111.0 (103.0;116.0) **b, C**	0.001	115.0 (100.0;122.5) **c, B**	105.0 (101.5;108.5) **d, C**	0.015	111,0 (96.0;124.0) **e, B**	102.0 (96.0;106.00) **e, C**	0.166	0.043	0.745
45	136.0 (129.0;142.0) **a, A**	119.0 (98.0;123.0) **b, C**	0.008	122.0 (112.5;130.0) **c, A**	118.0 (113.0;120.0) **d, C**	0.023	105,0 (96,0;112,0) **e, B**	105.5 (103.0;115.0) **f, C**	<0.001	0.039	0.945
60	123.0 (111.0;136.0) **a, A**	106.0 (93.0;115.0) **b, C**	0.008	118.0 (106.0;120.0) **c, A, B**	111.0 (105.0;120.0) **d, C**	0.023	100,0 (97.0;110.0) **e, B**	105.0 (99.0;116.0) **f, C**	<0.001	0.012	0.848
90	120.0 (107.0;128.0) **a, A**	98.0 (86.0;108.0) **b, C**	0.018	106.0 (101.0;109.0) **c, A, B**	103.5 (98.5;107.0) **c, C**	0.398	97,0 (90.0;105.0) **e, B**	98.0 (95.0;102.0) **e, C**	0.682	0.026	0.906
120	98.0 (89.0;103.0) **a, A**	99.0 (91.0;105.0) **b, C**	0.008	93.0 (91.0;100.0) **c, A**	96.0 (90.0;98.0) **d, C**	0.023	94,0 (90.0;97.0) **e, A**	94.0 (85.0;96.0) **f, C**	<0.001	0.998	0.683
area under the curve (j²)	1215.0 (645.0;2527.5) **a, A**	806.2 (577.5;1320.0) **b, C**	0.003	2745.0 (2235.0;3135.0) **c, B**	2321.2 (1777.5;2707.5) **d, D**	0.002	1785.0 (1297.5;2092.5) **e, A**	1650.0 (1125.0;2130.0) **e, C**	0.078	0.003	0.002

Values represent the median of the variables in each group, values provided in brackets represent values for the 25th and 75th quartile in a given group (Q1;Q3); **a, b** – significant differences in the glycaemic index (GI) between traditional and partly modified meals in human adults with a BMI < 18.5 kg/m²; **c, d** – significant differences in the GI between traditional and partly modified meals in human adults with a BMI 18.5–24.9 kg/m²; **e, f** – significant differences in the GI between traditional and partly modified meals in human adults with a BMI ≥ 25.0 kg/m²; **A, B** – significant differences in the GI between

traditional meals in human adults with different BMI values; **C, D** – significant differences in the GI between partly modified meals in human adults with different BMI values; T – traditional version meals, M – modified version meals, *p*-BMI T/*p*-BMI M – *p*-value of traditional/modified versions of meals after consumption by persons with different BMI.

Table S3. Blood glucose concentration (mg/dL) of the study participants within 2 h after the consumption of curd cheese dumplings prepared according to the traditional and partly modified recipes.

**Time (min)**	**BMI < 18.5 kg/m² (*n*=12)**			**BMI 18.5–24.9 kg/m² (*n*=12)**			**BMI ≥ 25.0 kg/m² (*n*=11)**			***p* BMI T**	***p* BMI M**
	**T**	**M**	***p***	**T**	**M**	***p***	**T**	**M**	***p***		
0	87.5 (86.0;94.0) **a, A**	91.0 (86.0;94.0) **b, C**	0.004	77.0 (73.0;85.0) **c, B, A**	81.0 (81.0;85.0) **d, D, C**	0.003	84.0 (79.0;92.0) **e, A**	83.0 (76.0;91.0) **f, C**	0.003	0.014	0.016
15	100.0 (96.0;108.5) **a, A**	103.0 (89.0;109.0) **b, C**	0.004	101.0 (87.0;111.0) **c, A**	85.0 (85.0;92.0) **d, C**	0.003	95.0 (86.0;103.0) **e, A**	90.0 (84.0;109.0) **f, C**	0.003	0.794	0.630
30	116.5 (104.5;120.0) **a, A**	107.0 (105.0;118.0) **a, C**	0.583	105.0 (99.0;125.0) **c, A**	106.0 (94.0;109.0) **c, C**	0.499	107.0 (101.0;127.0) **e, A**	106.0 (96.0;120.0) **e, C**	0.095	0.981	0.662
45	106.0 (101.0;123.5) **a, A**	102.0 (97.0;111.0) **b, C**	0.004	105.0 (101.0;115.0) **c, A**	110.0 (102.0;117.0) **d, C**	0.003	104.0 (96.0;120.0) **e, A**	103.0 (95.0;118.0) **f, C**	0.003	0.982	0.914
60	100.5 (96.0;106.0) **a, A**	98.0 (90.0;102.0) **b, C**	0.004	103.0 (98.0;108.0) **c, A**	98.0 (90.0;108.0) **d, C**	0.003	104.0 (99.0;131.0) **e, A**	101.0 (97.0;106.0) **f, C**	0.003	0.912	0.997
90	95.0 (92.0;99.0) **a, A**	95.0 (90.0;98.0) **a, C**	0961	96.0 (90.0;102.0) **c, A, B**	88.0 (82.0;95.0) **d, C, D**	0.007	104.0 (94.0;128.0) **e, B**	98.0 (95.0;106.0) **e, C**	0.122	0.036	0.009
120	89.0 (84.0;94.5) **a, A**	90.0 (85.0;90.0) **a, C**	0.702	87.0 (84.0;94.0) **c, A**	91.0 (86.0;93.0) **c, C**	0.624	94.0 (92.0;117.0) **e, A**	91.0 (84.0;100.0) **e, C**	0.147	0.906	0.959
area under the curve (j²)	1061.2 (892.5;1391.2) **a, A**	660.0 (570.0;1065.0) **b, C**	0.002	2118.7 (1668.7;3630.0) **c, B**	1545.0 (1143.7;2025.0) **d, D**	0.002	2212.5 (1267.5;2752.5) **e, B**	1770.0 (1125.0;2182.5) **f, D**	0.002	0.003	0.003

Footnote as in Table 3.

Table S4. Blood glucose concentration (mg/dL) of the study participants within 2 h after the consumption of pancakes with curd cheese prepared according to the traditional and partly modified recipes.

**Time (min)**	**BMI < 18.5 kg/m^2 (*n*=10)**			**BMI 18.5–24.9 kg/m^2 (*n*=12)**			**BMI ≥ 25.0 kg/m^2 (*n*=15)**			***p* BMI T**	***p* BMI M**
	**T**	**M**	***p***	**T**	**M**	***p***	**T**	**M**	***p***		
0	81.0<br>(78.0;85.0)<br>**a, A**	82.5<br>(78.0;90.0)<br>**a, C**	0.716	82.0<br>(75.5;85.0)<br>**c, A**	81.5<br>(78.5;86.0)<br>**c, C**	0.836	82.0<br>(78.0;92.0)<br>**e, A**	87.0<br>(80.5;94.0)<br>**e, C**	0.356	0.977	0.948
15	88.5<br>(86.0;92.0)<br>**a, A**	89.0<br>(82.0;94.0)<br>**b, C**	0.023	92.0<br>(88.0;99.5)<br>**c, A**	89.0<br>(85.5;95.0)<br>**d, C**	0.001	91.0<br>(84.0;96.0)<br>**e, A**	91.0<br>(87.0;97.5)<br>**f, C**	<0.001	0.986	0.988
30	98.0<br>(95.0;107.0)<br>**a, A**	98.5<br>(90.0;102.0)<br>**b, C**	0.023	108.5<br>(102.5;122.5)<br>**c, A**	98.5<br>(90.0;105.5)<br>**d, C**	0.001	102.0<br>(92.0;110.0)<br>**e, A**	111.0<br>(100.0;118.0)<br>**f, C**	<0.001	0.976	0.949
45	105.5<br>(101.0;107.0)<br>**a, A**	100.0<br>(93.0;109.0)<br>**a, C**	0.711	110.0<br>(106.0;122.5)<br>**c, A**	112.0<br>(102.0;115.5)<br>**c, C**	0.691	108.0<br>(96.0;115.0)<br>**e, A**	113.0<br>(107.5;121.0)<br>**e, C**	0.215	0.625	0.979
60	102.0<br>(96.0;103.0)<br>**a, A**	94.5<br>(87.0;105.0)<br>**b, C**	0.023	104.0<br>(101.0;108.0)<br>**c, A**	98.5<br>(90.5;107.0)<br>**d, C**	0.001	103.0<br>(100.0;114.0)<br>**e, A**	102.0<br>(93.5;113.0)<br>**f, C**	<0.001	0.896	0.997
90	95.0<br>(91.0;97.0)<br>**a, A**	91.0<br>(80.0;95.0)<br>**a, C**	0.519	97.5<br>(94.0;101.0)<br>**c, A**	93.0<br>(89.0;100.5)<br>**c, C**	0.473	98.0<br>(94.0;113.0)<br>**e, A**	98.5<br>(91.0;102.0)<br>**e, C**	0.383	0.892	0.982
120	89.5<br>(85.0;97.0)<br>**a, A**	86.5<br>(82.0;98.0)<br>**b, C**	0.023	92.5<br>(90.0;101.0)<br>**c, A**	96.5<br>(83.5;100.0)<br>**d, C**	0.001	96.0<br>(91.0;113.0)<br>**e, A**	93.0<br>(89.0;97.0)<br>**f, C**	<0.001	0.982	0.998
area under the curve (j^2)	1770.0<br>(1080.0;1987.5)<br>**a, A**	1102.5<br>(345.0;1912.5)<br>**b, C**	0.003	2336.2<br>(1728.8;2550.0)<br>**c, B**	1571.2<br>(1297.5;2310.0)<br>**d, C**	0.002	1995.0<br>(1237.5;2617.5)<br>**e, A**	1447.5<br>(1027.5;2580.0)<br>**f, C**	0.004	0.003	0.758

Footnote as in Table 3.

Table S5. Glycaemic indices of meals prepared according to the traditional and partly modified recipes.

**Meal type**	**BMI < 18.5 kg/m² (*n*=10)**			**BMI 18.5–24.9 kg/m² (*n*=12)**			**BMI ≥ 25.0 kg/m² (*n*=15)**			***p* BMI T**	***p* BMI M**
	**T**	**M**	***p***	**T**	**M**	***p***	**T**	**M**	***p***		
dumplings with potatoes and curd cheese stuffing	56.10 (51.08;56.43) **a, A**	22.45 (14.70;36.24) **b, C**	0.013	51.69 (50.80;71.68) **c, A**	46.55 (41.75;61.28) **d, D, C**	0.023	44.46 (23.53;53.94) **e, A**	39.23 (28.81;48.41) **e, C**	0.579	0.999	0.014
curd cheese dumplings	41.97 (30.16;58.54) **a, A**	25.21 (10.64;30.93) **b, C**	0.001	61.03 (54.35;73.80) **c, A**	34.35 (30.80;43.20) **d, C**	0.004	44.68 (26.95;56.68) **e, A**	34.26 (30.05;44.16) **e, C**	0.070	0.852	0.909
pancakes with curd cheese	43.25 (32.57;71.91) **a, A**	23.27 (14.62;38.12) **b, C**	0.003	53.41 (36.48;70.46) **c, A**	41.22 (34.04;48.85) **d, C**	0.001	39.20 (34.52;54.81) **e, A**	37.46 (20.56;49.71) **f, C**	<0.001	0.912	0.709

Footnote as in Table 3.
